# Rapid tests for HIV type discrimination in West Africa may perform differently

**DOI:** 10.7448/IAS.18.1.19460

**Published:** 2015-01-20

**Authors:** Bo L Hønge, Sanne Jespersen, Jens S Olesen, Christian Erikstrup, Christian Wejse

**Affiliations:** 1 Bandim Health Project, INDEPTH Network, Bissau, Guinea-Bissau; 2 Department of Infectious Diseases, Aarhus University Hospital, Aarhus, Denmark; 3 Department of Clinical Immunology, Aarhus University Hospital, Aarhus, Denmark; 4 GloHAU, Center of Global Health, School of Public Health, Aarhus University, Aarhus, Denmark

In the short report by Tchounga *et al*. [1], the performance of two rapid tests (Genie II HIV-1/HIV-2 (BioRad) and SD Bioline HIV-1/2 3.0 (Standard Diagnostics)) for HIV type discrimination was evaluated. The authors confirmed 85.7% of the initial HIV-2 and 23.0% of the HIV-1/2 dually reactive patients’ HIV type. We appreciate the authors’ efforts to illuminate difficulties in HIV type discrimination – this is important in West Africa, where HIV-1, HIV-2 and HIV-1/2 dual infections are prevalent. Yet, we find that certain aspects of their study need clarification.

First, the authors combined results from the two rapid tests to compare initial type diagnosis with the confirmatory tests. But as presented in a study from Guinea-Conakry by Chaillet *et al*. [2], the two rapid tests have different HIV type discriminatory capabilities. When compared with a diagnostic algorithm, Genie II HIV-1/HIV-2 identified 99.5% of HIV-1 samples, 95.2% of HIV-2-infected samples and 100% of HIV-1/2 dually reactive samples whereas SD Bioline HIV-1/2 3.0 identified 65% HIV-1, 69% HIV-2 and 100% of HIV-1/2 dually reactive samples. In Guinea-Bissau, SD Bioline HIV-1/2 3.0 has been used for HIV type discrimination for several years [3,4]. There we have described that SD Bioline HIV-1/2 3.0 was much less accurate than the next generation Genie test, the Genie III HIV-1/HIV-2 [5]. The reason may be that the test is hard to interpret, and we have described a high inter-observer variation (agreement 92.9%) of SD Bioline HIV-1/2 3.0 results, particularly among HIV-1/2 dual reactive results (agreement 62.2%) [6]. Because of different HIV type discriminatory capabilities of the tests, it would have been interesting to see the results of each test separately, and we hope the authors will share this information.

Second, the authors were only aware of the initial test type in 68.2% of the patients. Apparently, Genie II HIV-1/HIV-2 was used for 66.8% patients in Côte d'Ivoire and 50.0% in Mali while SD Bioline HIV-1/2 3.0 was used for 63.8% in Burkino Faso. When a high proportion of tests were of unknown type, it is uncertain which tests the poor performance refers to. We therefore propose a sensitivity analysis in which it is presumed that the 31.8% unknown tests were all either Genie II HIV-1/HIV-2 or SD Bioline HIV-1/2 3.0.

Third, the authors included patients who had initial HIV type determined during 2009–2012. Due to discolouration of the test window where results are read in the SD Bioline HIV-1/2 3.0 test, WHO recommended in January 2012 that national authorities should discontinue all affected lots of the product and any pending procurements should be cancelled [7]. Later SD Bioline HIV-1/2 3.0 was, however, reapproved by WHO [8], but invalid tests may have been used in the study and, if so, the authors should state that.

In conclusion, the paper by Tchounga *et al*. describes an important problem in West Africa that HIV discriminatory rapid tests may be inaccurate, but there are large differences between the performance of different rapid tests, and tests should preferably be evaluated separately.
